# Healthy knee asymmetry is a potential risk factor for knee osteoarthritis: Data from the osteoarthritis initiative

**DOI:** 10.1016/j.ostima.2025.100385

**Published:** 2025-11-21

**Authors:** Mikael J. Turunen, Alexander Paz, Lauri Stenroth, Santtu Mikkonen, Mimmi K. Liukkonen, Mika E. Mononen

**Affiliations:** a Department of Technical Physics, University of Eastern Finland, Kuopio, Finland; b Science Service Center, Kuopio University Hospital, Wellbeing Services County of North Savo, Kuopio, Finland; c Department of Clinical Radiology, Kuopio University Hospital, Wellbeing Services County of North Savo, Kuopio, Finland; d Department of Environmental and Biological Sciences, University of Eastern Finland, Kuopio, Finland

**Keywords:** Predicting, Development, Risk, Joint, Asymmetry

## Abstract

**Objective:**

Commonly used grading systems in knee osteoarthritis (OA) evaluation provide an overview of the disease severity with limited prognostic ability. Recently, efforts towards automated and objective deep- and machine-learning, and computational modeling-based prediction tools have been made, but they are complex and lack interpretability. This study aimed to identify knee joint morphology measures that can be easily quantified from plain radiographs and are indicative of the risk of radiographic OA development among subjects without definite radiographic OA, focusing especially on the asymmetry of the knees.

**Materials and Methods:**

Knee joint dimensions and angles were measured from anterior-posterior weight-bearing knee radiographs at baseline and 8-year follow-up time point. The subjects were grouped based on Kellgren-Lawrence grades at the 8-year follow-up and compared with regard to the knee joint dimensions and angles and their asymmetries between the subjects’ knees.

**Results:**

Absolute dimensions or angles at baseline were not associated with OA development. Instead, the asymmetry in the dimensions (relative difference between the left and right knee), was higher in subjects who developed radiographic knee OA during 8-year follow-up. The medial joint space asymmetry was associated with the development of advanced knee OA when it was over 10 % (OR = 1.87) or 15 % (OR = 3.27).

**Conclusions:**

Medial joint space asymmetry between the left and right knee of over 10 % could be a potential risk factor for developing knee OA.

## Introduction

The Kellgren-Lawrence (KL) grading system [1] is widely recognized as the standard radiological method for assessing knee osteoarthritis (OA). While it is considered the gold standard for evaluating OA severity through radiology, it was not designed for prognostic evaluation. To address this limitation, numerous predictive methodologies have emerged [[2], [3], [4], [5], [6]] with Kokkotis et al. [7] providing a comprehensive review of such approaches. In optimal scenarios, predictive accuracy has reached receiver-operating-characteristic area-under-the-curve values between 0.7 and 0.8. These models often rely on advanced machine or deep learning techniques, necessitating multimodal datasets, including biomechanical movement analyses (see e.g., Kokkotis et al. [7]), and/or computational modeling frameworks [3,8]. These strategies typically demand substantial training data and computational resources in their current iterations. At present, there are no broadly accepted clinical tools evaluating OA risk in asymptomatic individuals, particularly those with KL grades of 0 or 1.

Given the widespread availability and lower cost of radiography compared to other imaging modalities, the ability to identify quantifiable measures from knee radiographs that can indicate an increased risk of OA development would be of significant interest to clinicians. Such measures could serve to identify individuals who are at a higher risk of developing OA and may benefit from early intervention. Generally, the risk factors for knee OA development are based on subject characteristics (such as high age, obesity, female sex, joint injury, and genetics) or knee morphology, such as abnormal patellar, tibial, femoral and meniscus morphology, or changes in their alignment [[9], [10], [11], [12], [13], [14], [15], [16]]. However, these morphological risk factors focus on one knee, while little attention has been paid to comparing differences between individuals’ knees. Asymmetry of the individuals’ knees adduction moment [17], leg muscle mass [18], and anatomic angles and dimensions [19] have been associated with knee OA. Knee asymmetry analysis could be one noteworthy option, since it can be assumed to consider the natural differences of knee size and shape within the population, e.g., joint space, which could improve the sensitivity of observing, e.g., early unilateral alterations.

Hence, the aim of this study was to identify straightforward quantifiable measures of the knee joint from the most commonly used clinical knee radiographs that indicate the risk of OA development, i.e., would have prognostic value. In particular, this study focuses on the asymmetry of the dimensions of the knee joints as a risk factor of OA development. To this end, joint dimensions and angles of healthy knees from anterior-posterior weight-bearing radiographs were extensively characterized at baseline and grouped according to radiographic OA severity at the 8-year follow-up.

## Materials and methods

**Fig. 1:**
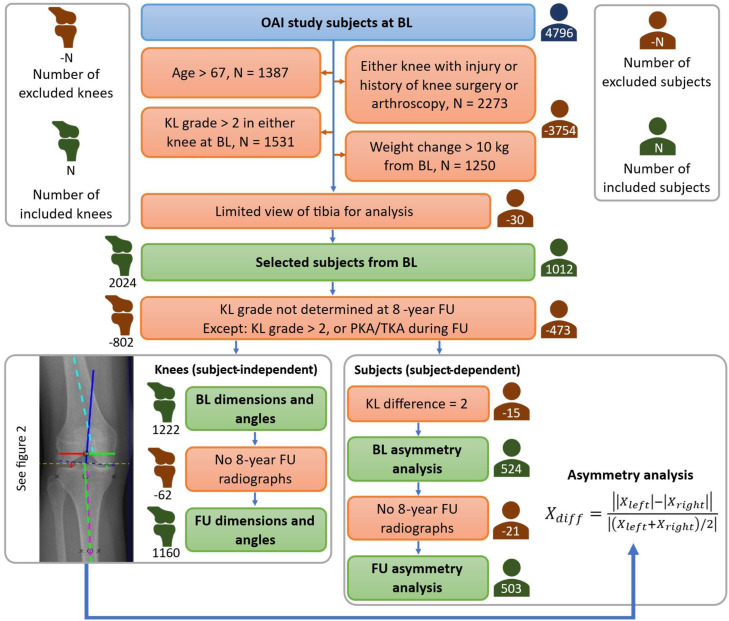
Exclusion criteria of the OAI subjects. The number of excluded (red) and included (green) subjects (person icon) and knees (knee icon) is shown next to each line. The first four exclusion criteria with overlapping subjects were pooled. The number of excluded subjects is shown with the exclusion criteria. Knee dimensions were measured for each included knee, but the asymmetry analysis was performed only for those subjects from whom both knees fulfilled the inclusion criteria. BL – baseline, FU – follow-up, PKA – partial knee arthroplasty, TKA – Total knee arthroplasty.

Data from the Osteoarthritis Initiative Database (OAI, https://nda.nih.gov/oai) which includes follow-up data of 4796 participants at baseline was used for this study. Fig. 1 shows the exclusion criteria for the subjects. Initial exclusion criteria included: age over 67 years, injury or history of knee surgery or arthroscopy, KL grade greater than 2 at baseline, and weight change of >10 kg at any time during the 8-year follow-up period. Due to the limited view of the proximal tibia in the radiographs, which hindered the measurement of all dimensions and angles (see *Measurement of dimensions and angles*), 30 subjects were further excluded. The dimensions and angles of 2024 knees (1012 subjects, 319 men and 693 women, see *Supplementary*) were measured. The measurements were done on baseline anteroposterior weight-bearing radiographs of both knees and grouped based on the individual knee KL grade (subject-independent) or the maximum KL grade of subjects’ both knees (subject-dependent) at the 8-year follow-up.

*Analysis of individual knees (subject-independent)*: All knees that fulfilled the inclusion criteria were included in the analysis of absolute knee dimensions and angles. The KL grade was not determined (in the OAI database) for every knee at the 8-year follow-up. Knees with no KL grade were excluded, leaving 1222 individual knees. Subjects and knees with severe OA (KL grade over 2 and those with partial or total knee arthroplasty; PKA/TKA) assessed at an earlier follow-up time point were not excluded, as it was assumed that the KL grade was static or increased. The knees were grouped according to the OA severity (KL grade) determined at the 8-year follow-up: KL01 (KL grades 0– 1), KL2 (KL grade 2), KL34S (KL grades 3–4, and PKA/TKA (Surgery)).

*Analysis of subject knees (subject-dependent)*: Evaluation of asymmetry requires data from both knees. Thus, the subjects who had a KL grade for only one knee (in the OAI database) were excluded. Additionally, subjects having a KL grade difference of 2 in their knees, i.e. KL0 and KL2, were excluded to limit the effect of KL grade difference on the asymmetry analysis (see *Measurement of dimensions and angles*), resulting in 524 included subjects. The subjects were grouped by OA severity (maximum KL grade of the two knees) at 8-year follow-up: KL01 (KL grades 0–1), KL2 (KL grade 2), KL34S (KL grades 3–4, and PKA/TKA (Surgery)).

*Comparison of baseline and 8-year follow-up dimensions and angles*: To assess changes in knee dimensions and angles over the 8-year follow-up for the included subjects and knees, those with anteroposterior radiographs taken at the 8-year follow-up were included in the analysis (see *Measurement of dimensions and angles* and *Statistical analysis*). Here, a total of 503 subjects and 1160 knees were included and grouped based on their 8-year follow-up KL grades. In the KL34S group, the knees with PKA/TKA (implant visible in the 8-year follow-up radiographs) were set to have zero joint space, since the joint space measured from the implant does not represent the natural joint space, and were excluded from the statistical analyses when comparing the 8-year follow-up data to the baseline (but not from the baseline-only analysis). Thus, one additional group (denoted as KL34) was formed that excluded the PKA/TKA knees. The 8-year follow-up data was compared with the baseline data.

*Stratification of knees and subjects based on the baseline KL grade*: In addition to the inclusion of baseline knees and subjects with baseline KL grade of 0–2, the grouping was also performed separately for KL01 and KL2 knees. For results, see Supplementary.

Ethical approval for collecting subject information was granted by the OAI. Weight-bearing radiographs (X-rays) were acquired under typical guidelines for annual and total radiation dosage to research subjects. Written consent was obtained from all subjects prior to each clinic visit. The OAI study was approved by the Institutional Review Board (IRB) for the University of California, San Francisco (UCSF), and its affiliates. The IRB approval was also obtained from all four clinical sites located at Brown University in Rhode Island, Ohio State University in Columbus, Ohio, University of Maryland/Johns Hopkins University joint center in Baltimore, Maryland, and at the University of Pittsburgh in Pennsylvania. Further details about the OAI data are accessible on the OAI website (https://nda.nih.gov/oai/).

### Measurement of dimensions and angles

**Fig. 2:**
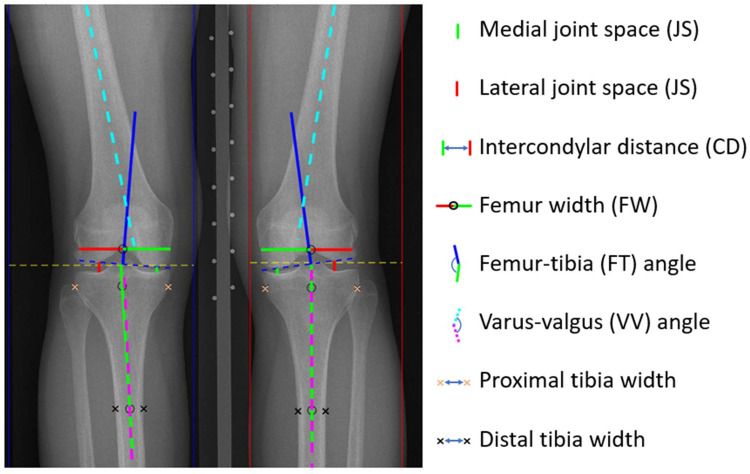
Dimensions and angles measured from the load-bearing anterior-posterior knee radiographs. The yellow horizontal dashed line was used for setting the approximated proximo-distal center of the tibiofemoral joint.

Joint dimensions and angles of the knees were analyzed from anterior-posterior weight-bearing knee radiographs using a custom-made Matlab (v. R2019b, MathWorks Inc., MA) graphical user interface (GUI) (Fig. 2). All measurements were performed blinded from KL-grade and subject sex by one researcher experienced in knee radiographs. In the GUI used for the analysis, custom-made automated scripts provide rough estimates of the locations for the measurements. First, it recognizes the two legs from the radiograph (blue and red boxes in Fig. 2)and then finds the joint spaces (yellow dashed lines in Fig. 2). Through vertical and lateral intensity profile estimations, it finds the rough locations of the 2-dimensional coordinates used in the measurements of the dimensions and angles. After the automated part, the user manually corrects (or approves) each set coordinate for both knees. For each measurement, the GUI provides a pop-up window with a magnification of the region to aid the user in positioning the measurement coordinate. The dimensions were measured in pixels and multiplied by the pixel size, which was obtained from the DICOM header of the radiograph. Each measurement was recorded to six decimal places.

Joint spaces were measured from the mediolateral centerline (visually estimated) of the medial and lateral condyles [20]. The intercondylar distance was defined as the distance between condyle centerlines. The femur width corresponds to the total width of the condyles at the location of the top of the intercondylar fossa. Femur-tibia angle was defined earlier [21] as the angle between the tibial axis (a line connecting the center of bone edges at a distance of 1 cm and 10 cm distal from the tibial plateau) and the femoral axis (perpendicular to a line tangent to the base of femoral condyles). Slightly deviating from the above-cited study, in this study the line tangent was based on the femoral coordinates of joint space measurements. Varus-valgus angle was measured as the angle between the anatomic axes of the tibia and femur. The proximal and distal tibia widths were the distances between the outer margins 1 cm and 10 cm, respectively, of the tibia from the tibial plateau. Fig. 2 depicts all the above-mentioned measures. The intra-rater variability, i.e., repeatability of the measurements by the same researcher who made the reported data, was evaluated with a subset of 25 randomly selected subjects (50 knees). The relative ( %) intra-rater variability for each dimension and angle is shown in Table 1.

**Table 1:** Intra-rater variability (and the absolute average difference) of the dimensions and angles.

Lateral JS	Medial JS	Intercondylar distance	Femur width	Proximal tibia width	Distal tibia width	FT angle	VV angle
4.96 % 0.31 mm	6.13 % 0.28 mm	1.62 % 0.79 mm	0.94 % 0.75 mm	1.26 % 0.97 mm	2.33 % 0.64 mm	6.39 % 0.38 °	17.02 % 0.79 °

Asymmetry was calculated as the relative differences *X_diff_* between the left and right knees for each dimension for each subject as

(1) $$X_{diff}=\frac{\left|\left|X_{left}\right|-\left|X_{right}\right|\right|}{\left|\left(X_{left}+X_{right}\right)/2\right|}$$

where *X_left_* and *X_right_* are the dimensions or angles measured from the left and right knees. Note that asymmetry is not knee- but subject-specific, i.e., it was evaluated only on subjects with both knees having a maximum KL grade of 2 at the baseline, and only when the KL grade difference was <2.

### Statistical analysis

The KL grouping was based on KL grades at the 8-year follow-up, based either on individual knee KL grades (average knee measure comparison) or the maximum KL grade of the two knees of a subject (asymmetry comparison). The group sizes (knees or subjects) for different analyses are presented in Fig. 1 (green boxes). Group comparisons for absolute dimensions, angles, and asymmetries were analyzed using linear mixed-effects models [22]. The same modelling framework was applied throughout to ensure methodological consistency across knee-level and person-level analyses. A mixed-effects procedure was chosen for calculation to allow maximum likelihood type estimation. Age, sex, weight, and height of the subjects were added as covariates in the model to account for their effect on the quantified measures. In knee-level cross-sectional analyses, measurements from both knees of the same participant could be included, creating a hierarchical data structure (knees nested within participants). This intra-subject correlation was accounted for by including appropriate random effects in the model. In longitudinal analyses that compared baseline and 8-year follow-up measurements for the same knee, the model accounted for both the correlation between knees within a participant and the correlation of repeated measurements over time within the same knee. In person-level analyses (e.g., asymmetry), only one observation per participant was included, and no nested structure was present; here the mixed-effects framework yields results equivalent to standard linear modelling. Note that the KL34S group includes knees with implants, which were set to have zero joint space, while the other measures represent the status of the knee with the implant. Thus, instead of the KL34S group, the KL34 was statistically compared to the KL01 and KL2 groups for the measures from the 8-year follow-up. Similarly, as for the above-mentioned anatomical parameters, age, height, and weight were grouped according to the KL grades at the 8-year follow-up. Instead of mixed-effects models, the Kruskal Wallis test with Dunn-Bonferroni correction for multiple tests was used to reveal statistical differences between the KL grades. The age, height, weight, absolute dimensions and angles, and asymmetries in the result figures are presented as mean ± 95 % confidence interval. The analyses were made with IBM SPSS Statistics software (v.29.0, IBM Corp., NY). For all statistical analyses, the level of significance was set at α = 0.05.

Odds ratios and 95 % confidence intervals [23] were calculated as unadjusted values. The odds ratios are presented without multivariable adjustment, as they were intended for descriptive purposes to illustrate associations between baseline asymmetry thresholds or established risk factors and OA outcome. Given the limited number of events in some subgroups, multivariable adjustment was not applied to avoid instability in the estimates. Odds ratios were calculated for medial joint space asymmetry (<10.0 %, ≥10.0 %, ≥12.5 %, and ≥15.0 % at baseline) and well-known risk factors or for knee OA with the following exposure statuses: sex (male or female), age (<50, 50–54, 55–59, and ≥60 years of age at baseline), body mass index (BMI [kg/m^2^]; 20 to <25, 25 to <30, and ≥30 at baseline). The outcome statuses were OA (KL34S) or no OA (KL01 or KL2). Odds ratio analysis for the subjects was included in the asymmetry analysis (n = 524, see Fig. 1).

It has to be noted that due to using age, sex, weight, and height as covariates in the statistical model, statistically significant differences were found although the absolute differences might be negligible. Further, it should be remembered that all the dimensions and angles were measured at the baseline, and the grouping was done based on the KL grades at the follow-up.

## Results

### Dimensions and angles measured at baseline and grouped by 8-year follow-up KL grades

**Fig. 3:**
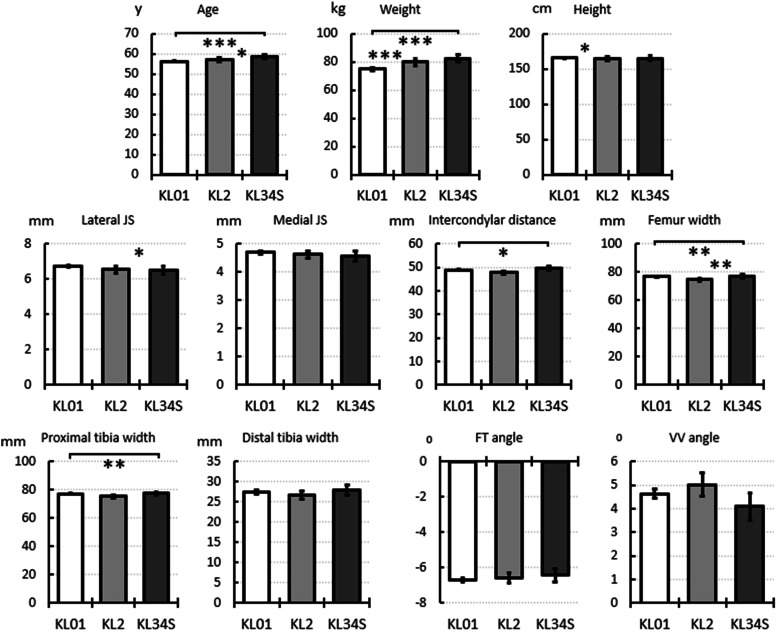
Demographic data and dimensions and angles at baseline grouped by 8-year follow-up grades. The bars indicate simple averages, and the error bars show the 95 % confidence intervals. Adjusted significant differences are indicated as * p < 0.05, ** p < 0.01, and *** p < 0.001, from the Kruskal-Wallis test with Dunn-Bonferroni correction (age, weight, height), or mixed-model analysis (dimensions and angles). y – years, FT – femur-tibia, VV – varus-valgus. For data in table form see Supplementary Table 3 and for box and whisker plots Supplementary Fig. 1.

The knee dimensions and angles did not differ markedly among the KL groups (Fig. 3) with statistically significant differences identified: the lateral joint space was smaller in the KL34S group than the KL2 group, the KL01 group had a smaller intercondylar distance than the KL34S, the femur width and proximal tibia width were larger in the KL34S group than the KL01 group, and femur width in the KL2 group was smaller than in the KL34S group. The varus-valgus and femur-tibia angles and distal tibia width did not differ between the groups. The age of the subjects was higher in the KL2 (< 1.8 %) and KL34S (< 4.3 %) groups compared to the KL01 group. Furthermore, the subjects in the KL2 (< 8.1 %) and KL34S (< 10.7 %) groups were heavier than the subjects in the KL01 group. See Supplementary Figure 3 for the same data including only KL01 graded knees at baseline and Supplementary Figure 4 for only KL2 graded knees.

### Knee asymmetry measured at baseline and grouped by 8-year follow-up KL grades

**Fig. 4:**
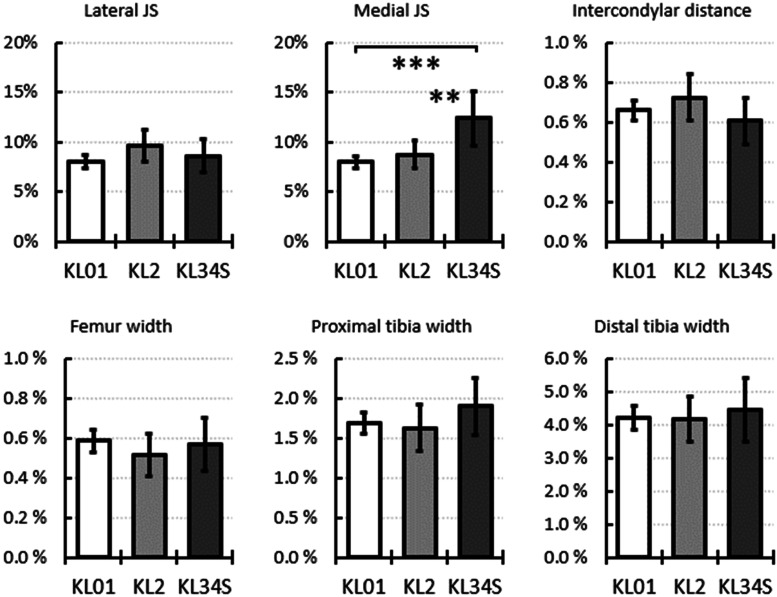
Relative differences, i.e., asymmetries, between left and right knee dimensions at baseline grouped by 8-year follow-up grades. The bars indicate simple averages, and the error bars show the 95 % confidence intervals. Adjusted significant differences are indicated as * p < 0.05, ** p < 0.01, and *** p < 0.001, from the mixed-model analysis. For data in table form see Supplementary Table 4 and for box and whisker plots Supplementary Fig. 2.

The medial joint space asymmetry was higher in the KL34S group than in the KL01 and KL02 groups. The asymmetries of the other dimensions were similar across all KL groups (Fig. 4). See Supplementary Figure 5 for the same data including only subjects with KL01 graded knees at baseline and Supplementary Figure 6 for only subjects with KL2 graded knees.

### Baseline vs 8-year follow-up dimensions and angles

**Fig. 5:**
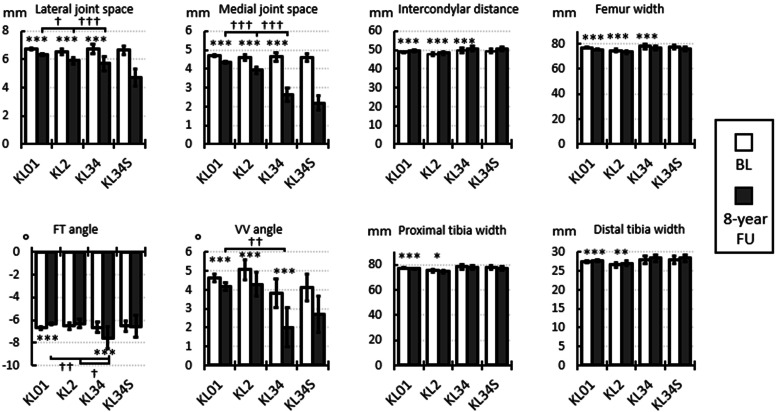
Dimensions and angles at baseline (BL) and 8-year follow-up (FU). KL groups are based on 8-year follow-up grades. The bars indicate simple averages, and the error bars show the 95 % confidence intervals. * Between baseline and follow-up measurements, † between KL grades for 8-year follow-up time point measurements (Figure 3 shows the statistics for the baseline measurements). Adjusted significant differences are indicated as * p < 0.05, ** p < 0.01, and *** p < 0.001, from the mixed-model analysis. FT – femur-tibia, VV – varus-valgus. For data in table form see Supplementary Table 5.

When comparing the baseline and 8-year follow-up dimensions and angles (Figure 5), the lateral and medial joint spaces decreased substantially in all KL groups. The intercondylar distance increased, while the femur width decreased in all KL groups. Proximal tibia width decreased, and distal tibia width increased in the KL01 and KL2 groups. However, these changes were relatively small (1–2 %) and their practical importance is negligible. The absolute follow-up femur-tibia angle was smaller in the KL01 group and larger in the KL34 group. The varus-valgus angle decreased during the follow-up in all groups compared to the baseline, with a maximum reduction of 47.5 % in the KL34 group.

When comparing the dimensions and angles at the 8-year follow-up of the KL groups (Fig. 5), the joint spaces decreased with increasing KL grade. In the KL34 group, the lateral and medial joint spaces were 15.5 % and 43.2 % lower, compared to the KL01 group. The absolute femur-tibia angle was larger in the KL34 group compared to the KL01 and KL2 groups, and the varus-valgus angle was smaller in the KL34 group than in the KL01 group. Intercondylar distance, femur width, and proximal tibia width did not differ across KL groups.

**Fig. 6:**
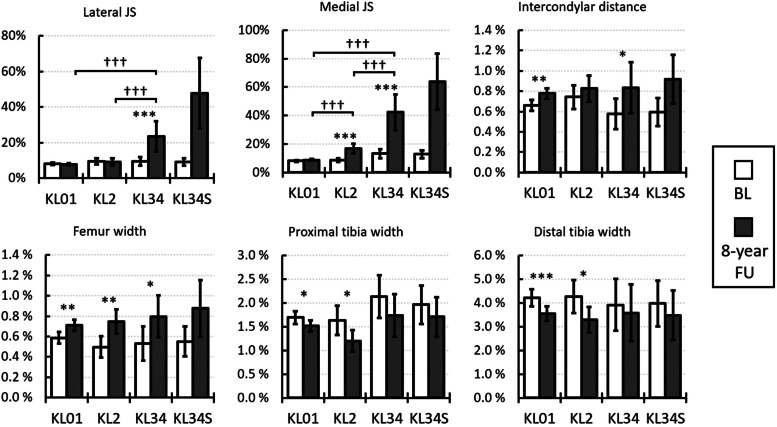
Relative differences i.e., asymmetries, between left and right knee dimensions at baseline (BL) and 8-year follow-up (FU). KL groups are based on 8-year follow-up grades. The bars indicate simple averages, and the error bars show the 95 % confidence intervals. * Between baseline and follow-up measurements, † between KL grades for 8-year follow-up time point measurements (Figure 4 shows the statistics for the baseline measurements). Adjusted significant differences are indicated as * p < 0.05, ** p < 0.01, and *** p < 0.001, from the mixed-model analysis. For data in table form see Supplementary Table 6.

The asymmetries in the lateral and medial joint spaces increased at the 8-year follow-up time point compared to the baseline, especially in the KL34 group (Fig. 6). An increase in the asymmetry was found also in the intercondylar distance and femur width, with differences in the intercondylar distance in the KL01 and KL34 groups, and in the femur width of all KL groups. The asymmetries in the proximal and distal tibia widths decreased in the KL01 and KL2 groups.

Lateral and medial joint space asymmetries increased with higher KL grades in the 8-year follow-up (Fig. 6). The asymmetries of the other dimensions did not differ statistically between KL grades, except for proximal tibia width, which was lower in the KL2 group compared to the KL01 and KL34 groups.

It has to be noted that no statistical analysis was done for the KL34S group (Fig. 5, Fig. 6). For the knees with knee replacement, joint spaces were set artificially at zero with the assumption of complete degeneration of the cartilage. However, this may not reflect the status of the joint prior to surgery.

### Osteoarthritis risk factors based on odds ratios

The odds ratios of associations between different sexes, ages, body mass indices, and medial joint space asymmetry values and advanced OA are shown in Table 2. Age over 60 years, BMI over 30 kg m^-2^, and medial joint space asymmetry over 10 % had odds ratios of over 1.0, indicating higher odds of advanced OA. Odds ratios for subjects including only KL01 graded knees are shown in Supplementary Table 7.

**Table 2:** Factors associated with advanced OA (KL34S).

**Variable**	**Category**	**OR (95 % CI)**
**Sex (vs Male)**	Female	1.35 (0.72 - 2.53)
**Age (years) (vs < 60)**	≥60	2.20 (1.24 - 3.89)
**BMI (kg m^-2^) (vs < 25)**	25 to <30	1.41 (0.79 - 2.51)
	≥30	2.22 (1.23 - 3.31)
**Medial JS asymmetry ( %)** **(vs < 10.0)**	≥10.0	1.87 (1.06 - 3.31)
	≥12.5	1.90 (1.04 - 3.47)
	≥15.0	3.27 (1.75 - 6.12)

Odds ratios (OR) with 95 % confidence intervals (CI) of associations between different sexes, ages, body mass indices (BMI), and medial JS asymmetry values and advanced OA (KL34S). OR reflects the odds of developing definite knee OA (KL34) during the 8-year follow-up for the exposed group (i.e., the category of a given variable) compared to the reference group (in parentheses).

## Discussion

In this study, easily measurable dimensions and angles from radiographs of healthy or doubtful OA knees (KL0-KL2) were analyzed and grouped based on OA severity at an 8-year follow-up. Data from the Osteoarthritis Initiative database was used, including demographic information, weight-bearing anterior-posterior knee radiographs, and the KL grades of the knees. The baseline dimensions and angles show scarce difference between knees that remained healthy and those that developed OA. However, the asymmetry of the dimensions between the left and right knee at a healthy baseline was associated with the development of knee OA, especially in the medial joint space. To our knowledge, this is the first study to assess the risk of knee OA development from simple anatomical measures derived from the most commonly used clinical anterior-posterior weight-bearing knee radiographs, without using deep- or machine-learning approaches.

A study by Nair et al. [19] analyzed the morphological variations – angular and linear measurements – from bilateral knee radiographs in a case-control study. The study identified five morphological features that were associated with increased risk of knee OA, three of them measured from skyline views of the patellofemoral compartment in 30 degrees flexion and two (larger proximal tibial tilt and varus malalignment) from the anterior-posterior views. The study included the analysis of symmetry of the morphologic measurements between the right and left knee. However, this was only done for the healthy controls and the asymmetry was not correlated to development of OA. Moreover, a case-control study cannot directly assess the predictive ability of the morphological variations when follow-up information is lacking. In this study, direct morphological measurements were not associated with development of OA, but the asymmetry between the subjects’ knees – derivative of the dimensions – does seem to be associated with OA development.

It is well known that joint space narrowing and knee alignment, among other risk factors and radiographic findings [24,25], are significantly associated with the severity of knee OA [21,[24], [25], [26], [27]]. However, joint space width (or articular cartilage thickness) measured at a single time point only describes the status of the knee and should be used as part of a broader OA diagnosis along with the other factors. In this study, the absolute baseline differences in dimensions and angles between knees without radiographic evidence of advanced OA (KL01) that later developed OA (KL34S) and those that remained KL01 over the 8-year follow-up were small. Some differences were statistically significant. As the repeated measures structure and potentially correlated observations were accounted for with the mixed-model structure in the analysis it is not likely that the differences are due to confounding effects. Still, we do not consider these findings clinically meaningful, since there are no specific thresholds for the measures in the diagnosis of OA, let alone for predictive purposes. Joint space narrowing is also age-related [28,29] which is supported by our findings: medial and lateral joint space decreased in the 8-year follow-up data compared to baseline in all KL groups. As depicted in the dimensions and angles measured from the 8-year follow-up radiographs, there are only minor (although statistically significant) differences between the KL grades, except for the notable reduction in medial joint space and the varus-valgus angle. The latter is most likely a consequence of the former. Furthermore, despite attempts to find a combination of measures (e.g., weighted sums of different combinations) that could distinguish between the KL01 and KL34S groups, no meaningful differences were found (data not shown).

However, the medial joint space asymmetry at baseline differed significantly between patients who developed radiographic OA and those who did not. On average, the asymmetry was ∼150 % larger in the KL34S group compared to the KL01 group and ∼130 % larger compared to the KL2 group. Furthermore, a significant increase in the asymmetry of the joint space from the baseline to the 8-year follow-up was evident. It is possible that elevated asymmetry is a sign of ongoing disease development that does not yet fulfill the criteria for a higher KL grade. Regardless, the current results suggest that asymmetry between the left and right knee medial joint space is associated with the development of knee OA, and it might be an easy tool for an objective indicator of early signs of the disease, in contrast to the more subjective and time-consuming KL grading.

The results indicate that a medial joint space relative difference (asymmetry) over 10 % could be a risk factor for knee OA. Fig. 4 shows this when comparing asymmetries between the KL34S group and the KL01 and KL2 groups. In addition, Table 2 shows an odds ratio of 1.87 for the association between medial joint space asymmetry over 10 % and advanced OA, and an odds ratio of 3.27 when asymmetry is over 15 %. These odds ratios are comparable to or larger than those of sex (female: 1.35), age (≥60 years: 2.20), and body mass index (≥30 kg m^-2^: 2.22) (Table 2), which are generally considered the most important risk factors for OA. As for the joint space of the KL01 and KL2 knees, no difference was observed at the 8-year follow-up. However, the odds ratio analysis including only the KL01 subjects (see Supplementary Table 7) shows that the general trends persist, i.e., the estimated odds ratios for OA progression are higher for higher joint space asymmetry, and the odds ratios estimates are comparable to the analysis presented for KL0–2 grade baseline subjects (Table 2). However, confidence intervals of the odds ratios are obviously increased due to the lower sample size. Still, we consider this additional analysis to show that the phenomenon is not explained solely by the fact that the ones with asymmetry have more severe OA at baseline. Even if this were true, the results point towards medial joint space asymmetry being potentially a more sensitive measure reflecting future status than joint space itself or KL grade. Thus, asymmetry of the medial joint space, as a risk factor, may indicate the overall risk for knee OA.

One of the main benefits of a simple and accessible tool for knee OA risk assessment is that a variety of healthcare professionals can use it without requiring specialized training, computing power, or special equipment. This is particularly valuable in settings where access to specialized equipment or computational methods may be limited. This type of tool may also be more cost-effective than more complex approaches that require developing artificial intelligence or other advanced computational methods, making it a valuable tool for healthcare systems with limited budgets, and especially saving valuable working time. In addition, a simple risk assessment tool may be more acceptable to patients and healthcare providers, as it is more transparent and easier to understand than complex computational methods. Technically, asymmetry can be determined using any software for clinical imaging visualization with measurement capabilities, giving the method promising potential for reproducibility and for transferability.

There are some limitations in this study. First, excluding those over age 67 constrains the generalizability in higher ages. This threshold was chosen to include subjects with a maximum age of 75 at the 8-year follow-up to limit the effect of age as a risk factor for OA. Second, we excluded individuals with a history of knee injury or surgery and those whose weight changed >10 kg from baseline. These exclusions have implications for interpreting the results. Because individuals were excluded based on future weight change, the results cannot be interpreted as showing the prognostic value of the identified risk factor. Instead, the findings, e.g., asymmetry in joint space, should be viewed as potential prognostic risk factors and their prognostic value should be confirmed in future studies. Excluding individuals based on knee injury or surgery also limits the generalizability of the findings. Future studies should investigate whether joint space asymmetry is also a risk factor for knee OA in individuals with knee injury or surgery. Knee injuries have been reported to lead to the development of joint space asymmetry [30] in some individuals, but it is currently unknown if the asymmetry is associated with knee OA risk. Third, exclusion of the subjects with KL grade difference of 2 (15 subjects) between the knees is likely to reduce the bias of the results compared to if these subjects had been included. The authors believe that by excluding the presumably high-risk subjects, inclusion in this study was conservative and strengthens the reliability of the results.

There are also limitations arising from the data collection in the OAI database. The OAI database includes only individuals aged 45 years and older, constraining the generalizability of the findings to younger populations. Although the prevalence of OA is low in people under 45 years, including younger subjects would strengthen the study. In addition, the OAI database is primarily Caucasian, and the results may not apply to other ethnic groups [31]. Furthermore, the orientation of the knees in the radiograph projections affects the apparent dimensions and angles measured. However, the large sample size of this study is likely to have minimized the impact of such errors. The OAI systematically excluded participants who were unlikely to experience progression (see Supplementary for criteria) and thus did not have KL grades assessed at the 8-year follow-up. Overall, this systematic exclusion most likely reduces the number of healthy (KL01 group) subjects included in this study. Without this exclusion, the ratio of healthy to OA subjects would most likely be higher than the ratio of subjects included in the current study (approximately 10 % with advanced OA at the 8-year follow-up). Although there is a possibility that the exclusion could introduce bias, the authors believe its effect on the conclusion is likely minimal.

Adding to the limitations, asymmetry may be used as a risk factor for subjects with radiographically healthy or doubtful OA knees (KL0-KL2), where the joint space narrowing is not prominent. In bilateral knee OA, the disease might progress at the same pace in both knees, and might not show an elevation in the asymmetry. Especially in the early phases of the disease development (KL 2 or lower), the asymmetry analysis would not indicate risk for OA. Also, evaluating risk for individuals with definite OA (KL3–4) would be pointless, and the asymmetry should not be utilized as a risk factor for future OA development. In the future, a combination of asymmetry and the absolute value of the joint space could provide a risk metric that also captures early bilateral changes in those without marked asymmetry. The dimensions and angles were measured by one researcher. For generalizability, it would be beneficial to evaluate the inter-rater variability. However, in this study, the main objective was to evaluate whether the asymmetry of the knees would be linked to OA. To minimize the effect of factors such as inter-rater variability, we chose to maximize the repeatability of the measurements. The intra-rater variability was generally low (Table 1). Work injuries or unreported traumas that might induce low-grade inflammation, influencing the risk of knee OA development [32] could not be included in the exclusion criteria. These risk-elevating features might also be present in future patients, and the asymmetry determination could prompt the clinician to inquire about these topics, improving the assessment of potential elevation of knee OA risk.

In conclusion, this study suggests that evaluating the asymmetry of knee dimensions may improve the assessment of the risk of radiographic knee OA development in healthy or early OA knees. Absolute knee measures from anterior-posterior radiographs in healthy and early OA stages appear not to be related to the follow-up KL grades, indicating that they are not suitable for evaluating the risk of OA development. Asymmetry analysis based on the relative differences between the dimensions of the left and right knee could be an easy and effective way to assess the risk of OA development and could be implemented in clinical practice by a variety of healthcare providers. Here, we identified asymmetry in the medial joint space, easily calculated with Eq. (1), with dimensions obtained with any typical clinical image viewer, as a potential predictor of future radiographic OA development. The data showed that asymmetry of >10 % could serve as an indicator of elevated risk. However, this needs to be investigated further. Future studies should seek to verify the current findings, expand the age range in the analyses to confirm whether the predictive ability of the joint space asymmetry depends on age, and attempt to define an optimal cut-off point for clinical use.

## Author contributions

*Mikael J. Turunen*: conceptualization, formal analysis, data curation, investigation, methodology, software, validation, visualization, writing - original draft, writing - review & wditing. *Alexander Paz*: data curation, validation, writing - review & editing. *Lauri Stenroth*: data curation, validation, writing - review & editing. *Santtu Mikkonen*: formal analysis, data curation, validation, writing - review & editing. *Mimmi K. Liukkonen*: data curation, validation, writing - review & editing. *Mika E. Mononen*: conceptualization, funding acquisition, methodology, software, project administration, writing - review & editing. All authors approve the final version to be published and agree to be accountable for all aspects of the work if questions arise related to its accuracy or integrity.

## Authors’ disclosures

Mikael J Turunen and Mika E Mononen own stock of Aikoa Technologies Oy. Other authors have nothing to disclose.

## Declaration of competing interest

The authors declare the following financial interests/personal relationships which may be considered as potential competing interests:

Mikael J. Turunen reports financial support was provided by Research Council of Finland. Mikael J. Turunen reports financial support was provided by Kuopio University Hospital. Mika E. Mononen reports financial support was provided by Research Council of Finland. Lauri Stenroth reports financial support was provided by Research Council of Finland. Santtu Mikkonen reports financial support was provided by Research Council of Finland. Mimmi K. Liukkonen reports financial support was provided by Kuopio University Hospital. Mika E. Mononen reports financial support was provided by Sigrid Jusélius Foundation. Alexander Paz reports financial support was provided by Finnish Cultural Foundation. Mikael J. Turunen reports a relationship with Aikoa Technologies Oy that includes: board membership and equity or stocks. Mika E. Mononen reports a relationship with Aikoa Technologies Oy that includes: board membership and equity or stocks. If there are other authors, they declare that they have no known competing financial interests or personal relationships that could have appeared to influence the work reported in this paper.

## Appendix Supplementary materials

Image, application 1
