# Supplementary material for: Healthy knee asymmetry is a potential risk factor for knee osteoarthritis: Data from the osteoarthritis initiative
Source: Osteoarthr Imaging. 2025 Nov 21;5(4):100385. doi: 10.1016/j.ostima.2025.100385 (PMC13228709; doi:10.1016/j.ostima.2025.100385)
Supplement: Supplementary file 1 [file mmc1.docx]

**Healthy Knee Asymmetry Is a Potential Risk Factor for Knee Osteoarthritis – Data from the Osteoarthritis Initiative**

**SUPPLEMENTARY MATERIAL**

**Systematic exclusion of subjects made in the OAI database**

Referring to the OAI document entitled “kXR_SQ_BU_Descrip.pdf” (downloadable from the OAI webpage), the KL grades at 8-year follow-up timepoint were done in project 37 (or 42):

“a. Any knees eligible to develop incident radiographic knee OA between the 48-month and 96-month visits i.e.: knees had 72-month and/or 96-month visit knee X-rays and were KLG 0 or 1 at baseline and did not have KLG≥2 with JSN≥1 on the 48-month X-ray (or the last time point with Project 15 data) to determine incidence by 96-months.

b. Any knees that had developed incident radiographic knee OAI between baseline and 48-month visits to assess worsening of disease over the next follow-up period (knees which were end stage prior to the 72-month visit were excluded).

c. A random selection of 246 knees which were KLG 2 or 3 at baseline to determine worsening of disease over the next follow-up period (knees which were end stage prior to the 72-month visit were excluded).”

**Supplementary Table 1.** Age, weight, height, and anatomical dimensions and angles of male and female subjects. The average ± standard deviations are shown. Individual samples t-test was used for statistical differences between male and female: ** p < 0.01.

|  | **Male (N = 319)** | **Female (N = 693)** |
| --- | --- | --- |
| **Age (y)** | 56.1 ± 6.3 | 57.1 ± 6.0 |
| **Weight (kg)** | 88.7 ± 13.6** | 72.2 ± 13.8 |
| **Height (cm)** | 173.4 ± 24.9** | 160.7 ± 18.4 |
| **Medial JS (mm)** | 5.25 ± 0.83** | 4.40 ± 0.74 |
| **Lateral JS (mm)** | 7.46 ± 1.16** | 6.29 ± 1.05 |
| **CD (mm)** | 54.2 ± 3.5** | 46.3 ± 2.9 |
| **CW (mm)** | 84.3 ± 5.0** | 72.6 ± 4.1 |
| **FT angle (°)** | -6.7 ± 1.8 | -6.5 ± 1.8 |
| **VV angle (°)** | 3.4 ± 2.9** | 5.2 ± 2.9 |
| **Prox. Tibia (mm)** | 84.8 ± 5.0** | 73.2 ± 4.5 |
| **Dist. Tibia (mm)** | 31.5 ± 3.3** | 25.7 ± 2.9 |

**Supplementary Table 2.** Dimensions and angles of right and left knees. The average ± standard deviations are shown. Paired samples t-test was used for statistical differences between left and right knee: * p < 0.05, ** p < 0.01.

|  | **Right (N = 1012)** | **Left (N = 1012)** |
| --- | --- | --- |
| **Medial JS (mm)** | 4.64 ± 0.86** | 4.71 ± 0.86 |
| **Lateral JS (mm)** | 6.79 ± 1.22** | 6.54 ± 1.20 |
| **CD (mm)** | 48.8 ± 4.8** | 48.7 ± 4.8 |
| **CW (mm)** | 76.3 ± 7.0* | 76.3 ± 7.0 |
| **FT angle (°)** | -7.1 ± 1.8** | -6.1 ± 1.7 |
| **VV angle (°)** | 4.8 ± 2.9* | 4.5 ± 3.1 |
| **Prox. Tibia (mm)** | 76.9 ± 7.1 | 76.8 ± 7.1 |
| **Dist. Tibia (mm)** | 27.6 ± 4.0** | 27.4 ± 4.1 |

**Supplementary Table 3.** Figure 3 numerical data in table form. Averages and [95% confidence intervals are shown.

| Fig 3 | Average [95% CI] | | |
| --- | --- | --- | --- |
|  | **KL01** | **KL2** | **KL34S** |
| Age (years) | 56.33 [55.93 - 56.72] | 57.01 [56.15 - 57.88] | 58.66 [57.67 - 59.65] |
| Weight (kg) | 74.8 [73.79 - 75.81] | 79.77 [77.43 - 82.12] | 82.43 [80.02 - 84.83] |
| Height (cm) | 165.26 [163.99 - 166.54] | 163.94 [161.21 - 166.66] | 165.08 [161.25 - 168.91] |
| Lateral JS (mm) | 6.73 [6.65 - 6.8] | 6.53 [6.33 - 6.73] | 6.51 [6.27 - 6.76] |
| Medial JS (mm) | 4.69 [4.64 - 4.75] | 4.61 [4.48 - 4.75] | 4.57 [4.4 - 4.74] |
| Intercondylar distance (mm) | 48.89 [48.59 - 49.2] | 47.74 [46.97 - 48.51] | 49.46 [48.58 - 50.34] |
| Femur width (mm) | 76.7 [76.25 - 77.14] | 74.76 [73.64 - 75.88] | 76.91 [75.7 - 78.12] |
| Proximal tibia width (mm) | 77.23 [76.78 - 77.69] | 75.3 [74.21 - 76.39] | 77.39 [76.16 - 78.62] |
| Distal tibia width (mm) | 27.44 [27.19 - 27.7] | 26.69 [26.06 - 27.32] | 27.9 [27.15 - 28.66] |
| FT angle (degrees) | -6.69 [-6.8 - -6.58] | -6.56 [-6.84 - -6.27] | -6.44 [-6.79 - -6.08] |
| VV angle (degrees) | 4.64 [4.45 - 4.83] | 5.02 [4.52 - 5.52] | 4.08 [3.52 - 4.65] |

**Supplementary Table 4.** Figure 4 numerical data in table form. Averages and ]95% confidence intervals] are shown.

| Fig 4 | Average [95% CI] | | |
| --- | --- | --- | --- |
|  | **KL01** | **KL2** | **KL34S** |
| Lateral JS | 8.05 % [7.38 % - 8.72 %] | 9.69 % [8.08 % - 11.29 %] | 8.6 % [6.92 % - 10.29 %] |
| Medial JS | 8.00 % [7.36 % - 8.65 %] | 8.74 % [7.35 % - 10.14 %] | 12.39 % [9.69 % - 15.09 %] |
| Intercondylar distance | 0.66 % [0.61 % - 0.71 %] | 0.72 % [0.61 % - 0.84 %] | 0.61 % [0.49 % - 0.72 %] |
| Femur width | 0.59 % [0.53 % - 0.65 %] | 0.52 % [0.41 % - 0.62 %] | 0.57 % [0.43 % - 0.7 %] |
| Proximal tibia width | 1.69 % [1.56 % - 1.83 %] | 1.63 % [1.34 % - 1.93 %] | 1.9 % [1.54 % - 2.26 %] |
| Distal tibia width | 4.22 % [3.87 % - 4.57 %] | 4.19 % [3.51 % - 4.88 %] | 4.46 % [3.48 % - 5.44 %] |

**Supplementary Table 5.** Figure 5 numerical data in table form. Averages and [95% confidence intervals] are shown.

| Fig 5 | | Average [95% CI] | | | |
| --- | --- | --- | --- | --- | --- |
|  |  | **KL01** | **KL2** | **KL34** | **KL34S** |
| Baseline | **Lateral JS (mm)** | 6.74 [6.67 - 6.82] | 6.52 [6.31 - 6.72] | 6.75 [6.43 - 7.07] | 6.65 [6.36 - 6.94] |
|  | **Medial JS (mm)** | 4.7 [4.65 - 4.75] | 4.61 [4.47 - 4.76] | 4.64 [4.43 - 4.86] | 4.6 [4.4 - 4.79] |
|  | **Intercondylar distance (mm)** | 48.94 [48.63 - 49.24] | 47.56 [46.81 - 48.31] | 49.9 [48.66 - 51.13] | 49.48 [48.35 - 50.61] |
|  | **Femur width (mm)** | 76.8 [76.35 - 77.24] | 74.57 [73.46 - 75.68] | 77.72 [76.01 - 79.43] | 77.16 [75.61 - 78.7] |
|  | **FT angle (degrees)** | -6.69 [-6.8 - -6.58] | -6.53 [-6.82 - -6.24] | -6.66 [-7.11 - -6.2] | -6.54 [-6.96 - -6.13] |
|  | **VV angle (degrees)** | 4.61 [4.42 - 4.8] | 5.07 [4.56 - 5.58] | 3.83 [3.1 - 4.56] | 4.1 [3.43 - 4.78] |
|  | **Proximal tibia width (mm)** | 77.32 [76.86 - 77.78] | 75.11 [74.03 - 76.19] | 78.13 [76.4 - 79.87] | 77.77 [76.21 - 79.32] |
|  | **Distal tibia width (mm)** | 27.49 [27.23 - 27.74] | 26.62 [25.98 - 27.25] | 27.96 [26.93 - 28.99] | 27.99 [27.08 - 28.9] |
| 8-year follow-up | **Lateral JS (mm)** | 6.31 [6.24 - 6.38] | 5.92 [5.7 - 6.13] | 5.7 [5.21 - 6.2] | 4.74 [4.16 - 5.33] |
|  | **Medial JS (mm)** | 4.33 [4.28 - 4.38] | 3.93 [3.77 - 4.1] | 2.64 [2.3 - 2.98] | 2.19 [1.85 - 2.54] |
|  | **Intercondylar distance (mm)** | 49.48 [49.19 - 49.77] | 48.44 [47.69 - 49.19] | 50.79 [49.7 - 51.88] | 50.32 [49.34 - 51.3] |
|  | **Femur width (mm)** | 75.3 [74.87 - 75.72] | 73.48 [72.43 - 74.54] | 76.47 [74.93 - 78.01] | 76.07 [74.64 - 77.51] |
|  | **FT angle (degrees)** | -6.34 [-6.45 - -6.23] | -6.3 [-6.68 - -5.92] | -7.57 [-8.5 - -6.64] | -6.56 [-7.47 - -5.66] |
|  | **VV angle (degrees)** | 4.18 [4 - 4.37] | 4.29 [3.7 - 4.89] | 2.01 [1.02 - 3] | 2.69 [1.78 - 3.6] |
|  | **Proximal tibia width (mm)** | 76.74 [76.31 - 77.17] | 74.67 [73.61 - 75.72] | 77.57 [75.97 - 79.18] | 77.27 [75.79 - 78.74] |
|  | **Distal tibia width (mm)** | 27.79 [27.54 - 28.04] | 27.05 [26.45 - 27.66] | 28.32 [27.39 - 29.25] | 28.4 [27.56 - 29.24] |

**Supplementary Table 6.** Figure 6 numerical data in table form. Averages and [95% confidence intervals] are shown.

| Fig 6 | | Average [95% CI] | | | |
| --- | --- | --- | --- | --- | --- |
|  |  | **KL01** | **KL2** | **KL34** | **KL34S** |
| Baseline | **Lateral JS** | 8.15 % [7.47 % - 8.83 %] | 9.40 % [7.75 % - 11.04 %] | 9.37 % [7.07 % - 11.67 %] | 9.19 % [7.21 % - 11.17 %] |
|  | **Medial LS** | 7.99 % [7.34 % - 8.65 %] | 8.53 % [7.14 % - 9.91 %] | 13.22 % [9.98 % - 16.47 %] | 12.7 % [9.86 % - 15.54 %] |
|  | **Intercondylar distance** | 0.66 % [0.61 % - 0.71 %] | 0.74 % [0.62 % - 0.86 %] | 0.58 % [0.43 % - 0.73 %] | 0.59 % [0.46 % - 0.73 %] |
|  | **Femur width** | 0.59 % [0.53 % - 0.64 %] | 0.50 % [0.39 % - 0.60 %] | 0.53 % [0.36 % - 0.70 %] | 0.55 % [0.40 % - 0.70 %] |
|  | **Proximal tibia width** | 1.69 % [1.55 % - 1.83 %] | 1.63 % [1.33 % - 1.94 %] | 2.13 % [1.68 % - 2.58 %] | 1.96 % [1.56 % - 2.37 %] |
|  | **Distal tibia width** | 4.21 % [3.85 % - 4.56 %] | 4.26 % [3.56 % - 4.97 %] | 3.91 % [2.83 % - 5.00 %] | 3.97 % [3.01 % - 4.93 %] |
| 8-year follow-up | **Lateral JS** | 7.87 % [7.16 % - 8.58 %] | 9.12 % [7.06 % - 11.17 %] | 23.58 % [15.09 % - 32.08 %] | 47.71 % [27.83 % - 67.59 %] |
|  | **Medial JS** | 8.84 % [8.02 % - 9.65 %] | 16.6 % [13.16 % - 20.03 %] | 42.37 % [29.73 % - 55.01 %] | 63.96 % [44.3 % - 83.62 %] |
|  | **Intercondylar distance** | 0.78 % [0.72 % - 0.83 %] | 0.83 % [0.70 % - 0.95 %] | 0.83 % [0.58 % - 1.09 %] | 0.92 % [0.68 % - 1.16 %] |
|  | **Femur width** | 0.71 % [0.65 % - 0.77 %] | 0.75 % [0.63 % - 0.87 %] | 0.80 % [0.59 % - 1.01 %] | 0.88 % [0.60 % - 1.15 %] |
|  | **Proximal tibia width** | 1.51 % [1.40 % - 1.63 %] | 1.20 % [0.97 % - 1.42 %] | 1.73 % [1.28 % - 2.19 %] | 1.71 % [1.29 % - 2.12 %] |
|  | **Distal tibia width** | 3.54 % [3.24 % - 3.85 %] | 3.29 % [2.75 % - 3.83 %] | 3.58 % [2.39 % - 4.78 %] | 3.48 % [2.45 % - 4.51 %] |

**Supplementary Table 7** Factors associated with advanced OA (KL34S), including only subjects with KL0-1 graded knees at baseline (excluding subjects with KL2 knees).

| **Variable** | **Category** | **OR (95% CI)** |
| --- | --- | --- |
| **Gender (vs Male)** | Female | 2.39 (0.89 - 6.44) |
| **Age (years) (vs < 60)** | ≥60 | 2.12 (0.97 - 4.62) |
| **BMI (kg m^-2^) (vs < 25)** | 25 to <30 | 1.08 (0.48 - 2.41) |
|  | ≥30 | 2.28 (1.03 - 3.38) |
| **Medial JS asymmetry (%)**  **(vs < 10.0)** | ≥10.0 | 1.54 (0.71 - 3.38) |
|  | ≥12.5 | 1.16 (0.48 - 2.82) |
|  | ≥15.0 | 2.32 (0.94 - 5.71) |

*Odds ratios (OR) with 95% confidence intervals (CI) of associations between different genders, ages, body mass indices (BMI), and medial JS asymmetry values and advanced OA (KL34S). OR reflects the odds of developing definite knee OA (KL34) during the 8-year follow-up for the exposed group (i.e., the category of a given variable) compared to the reference group (in parentheses)*


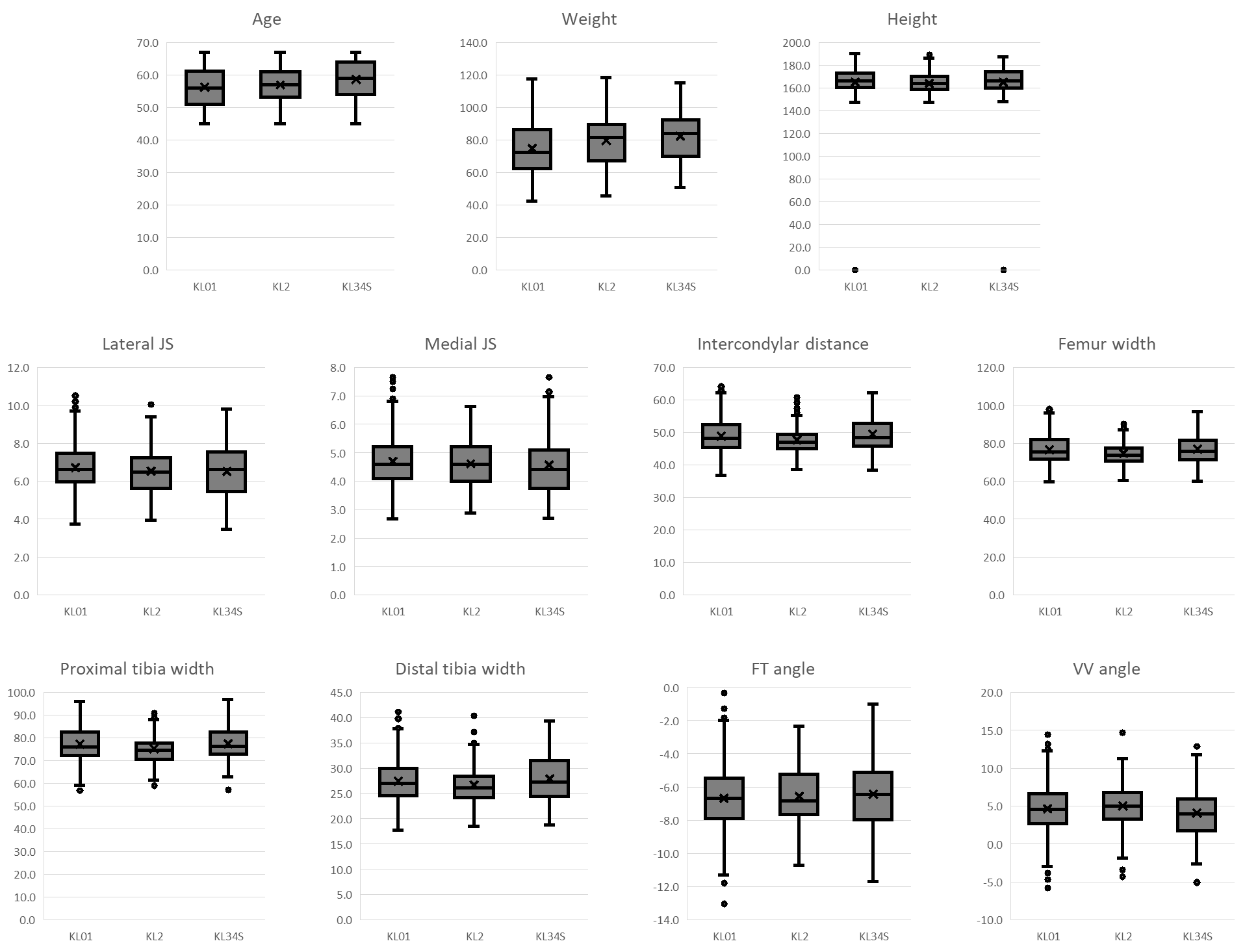


**Supplementary Figure 1.** Box and whisker of the data shown in Figure 3.


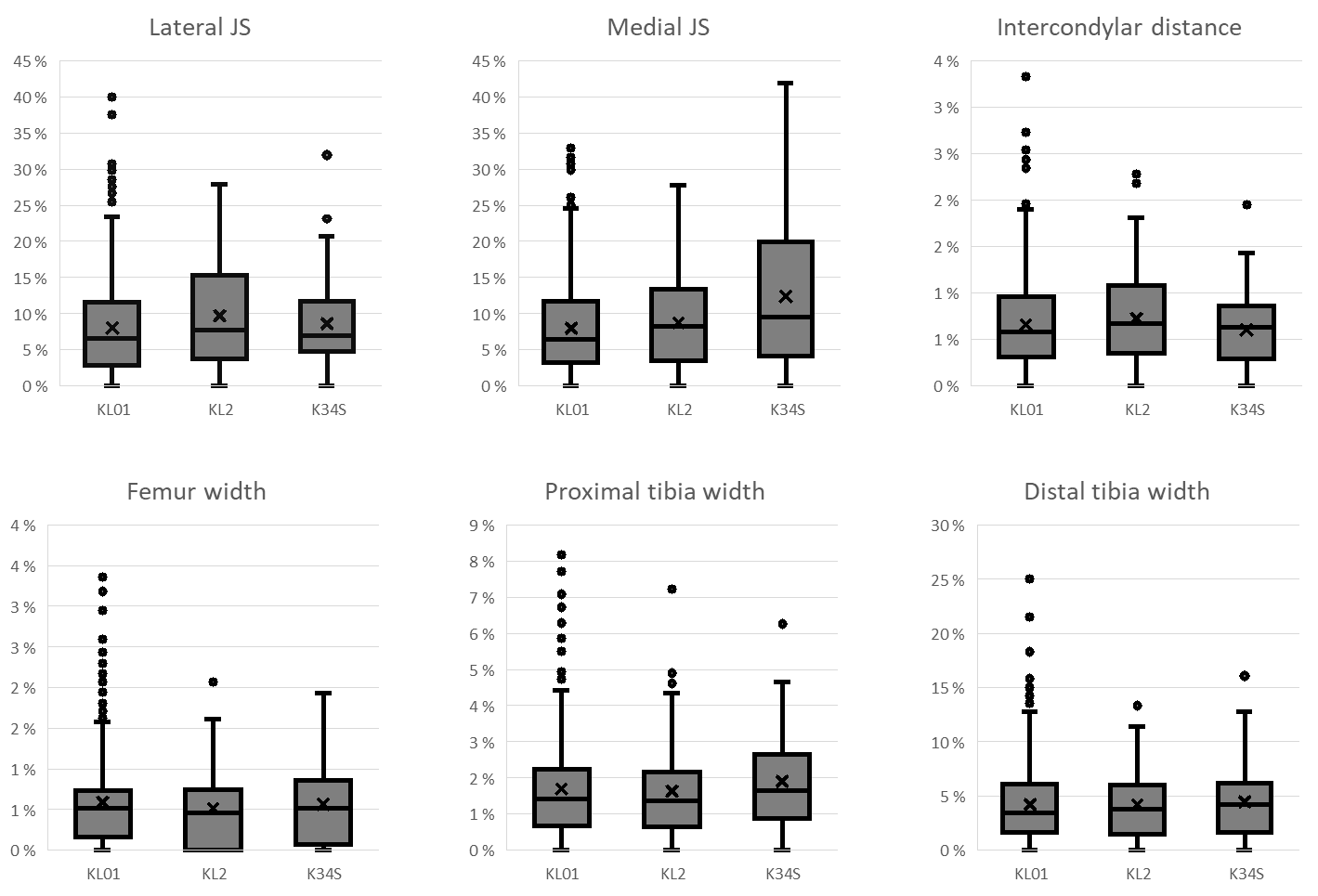


**Supplementary Figure 2.** Box and whisker of the data shown in Figure 4.


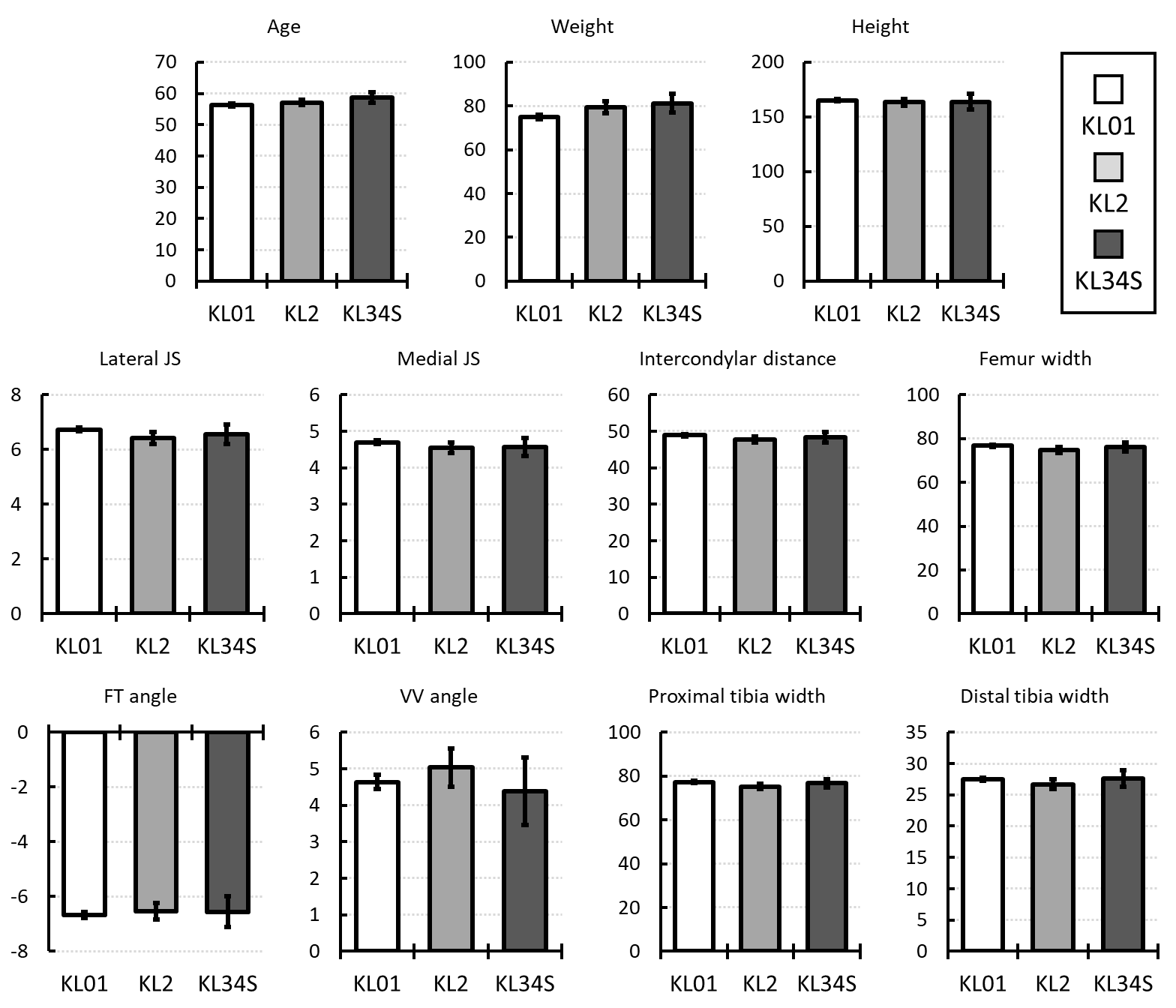


**Supplementary Figure 3.** Demographic data and dimensions and angles at baseline grouped by 8-year follow-up grades including only KL0-1 graded knees at baseline. The bars indicate simple averages, and the error bars show the 95% confidence intervals. y – years, FT – femur-tibia, VV – varus-valgus.


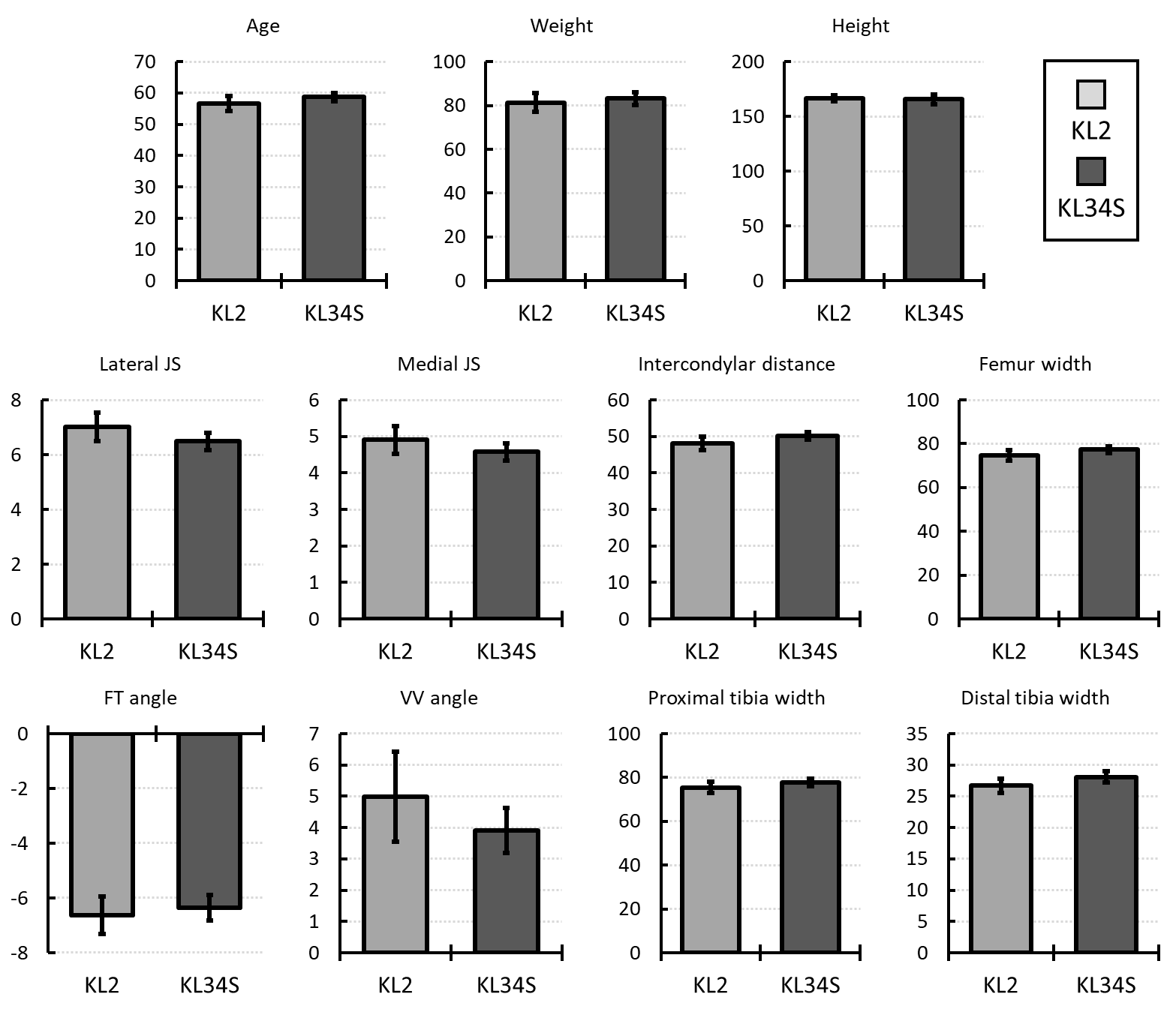


**Supplementary Figure 4.** Demographic data and dimensions and angles at baseline grouped by 8-year follow-up grades including only KL2 graded knees at baseline. The bars indicate simple averages, and the error bars show the 95% confidence intervals. y – years, FT – femur-tibia, VV – varus-valgus.


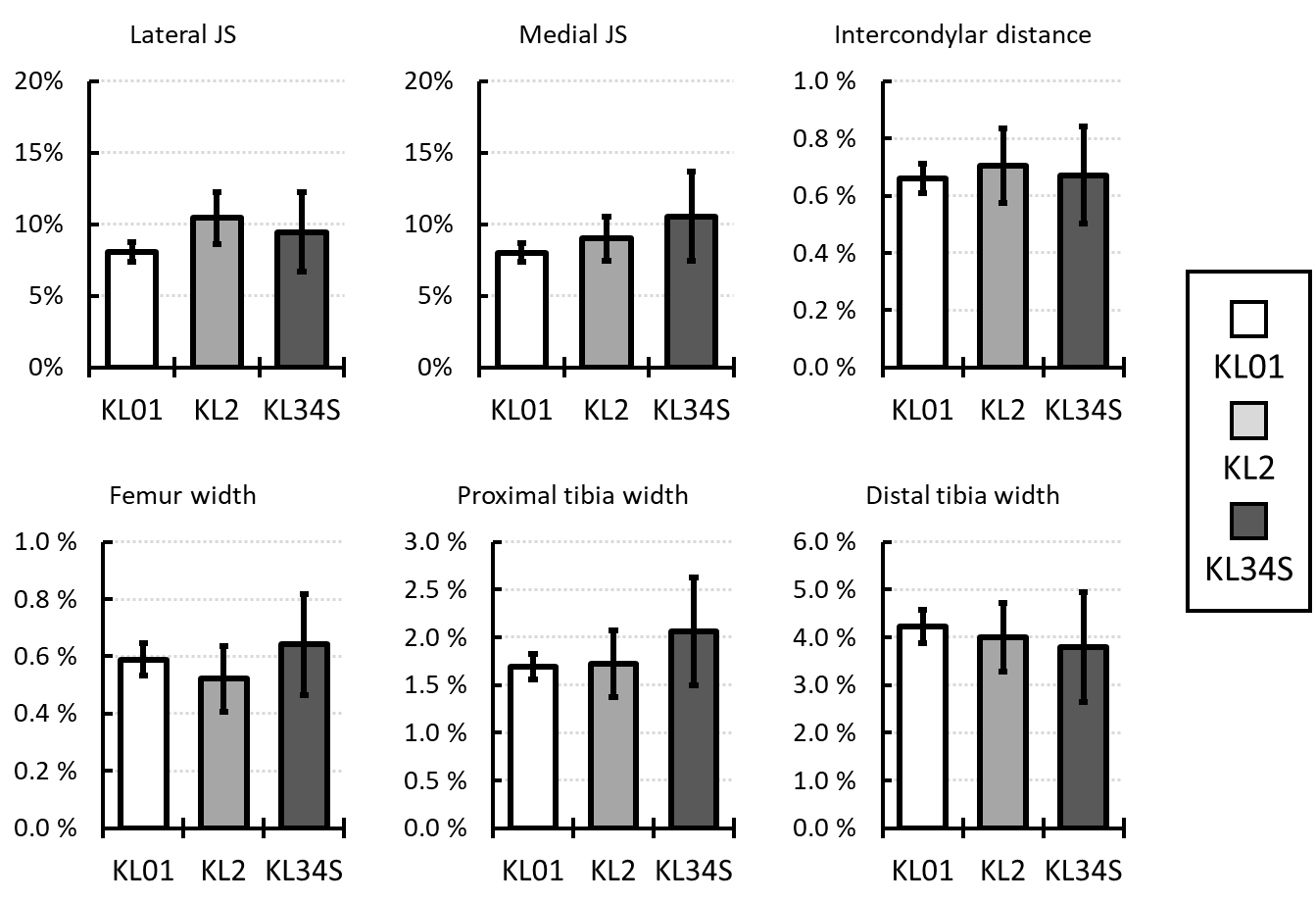


**Supplementary Figure 5.** Relative differences, i.e., asymmetries, between left and right knee dimensions at baseline grouped by 8-year follow-up grades including only subjects with KL0-1 graded knees at baseline. The bars indicate simple averages, and the error bars show the 95% confidence intervals.


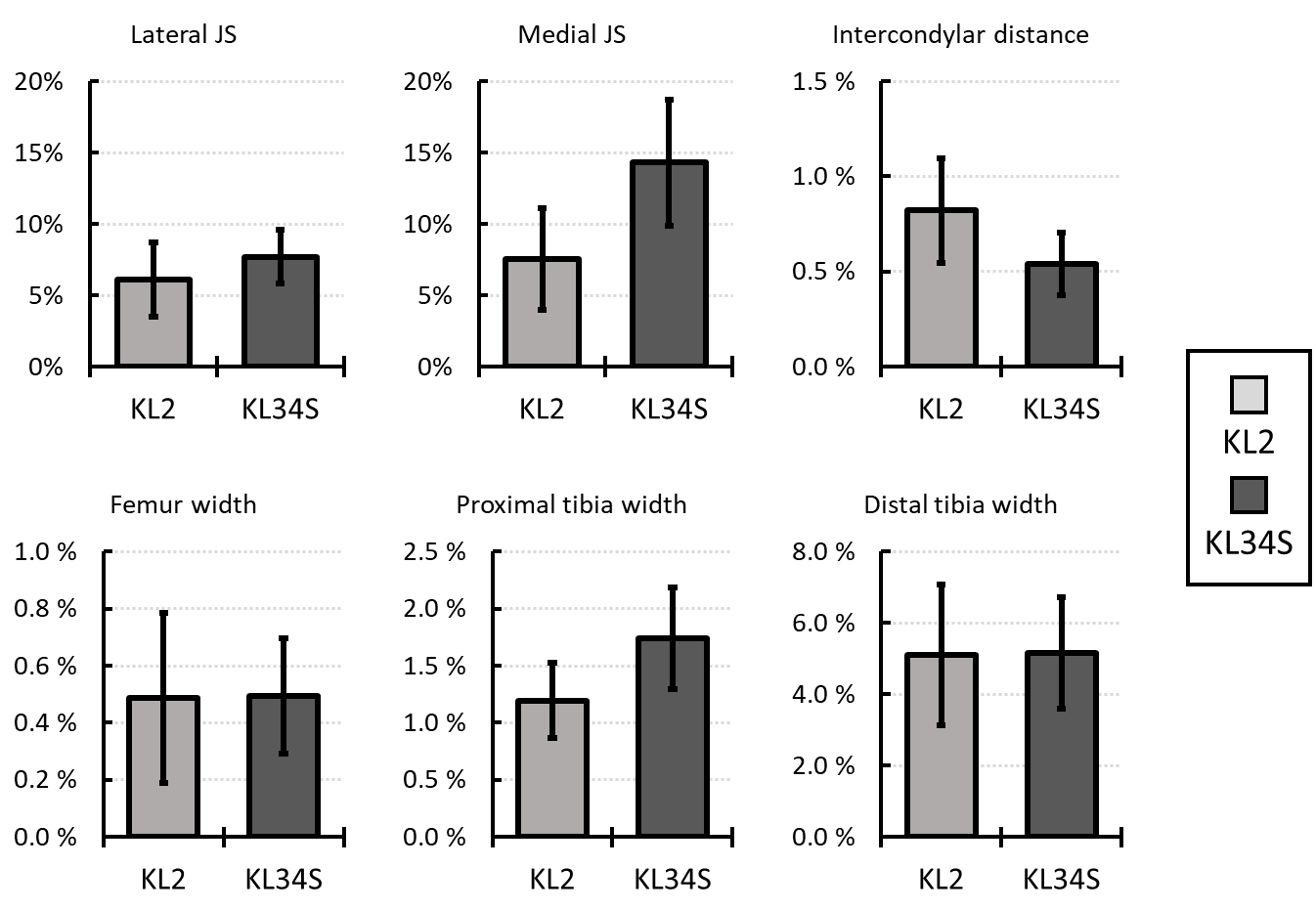


**Supplementary Figure 6.** Relative differences, i.e., asymmetries, between left and right knee dimensions at baseline grouped by 8-year follow-up grades including only subjects with KL2 graded knees at baseline. The bars indicate simple averages, and the error bars show the 95% confidence intervals.
